# Modeling the Impact of Bed-Net Use and Treatment on Malaria Transmission Dynamics

**DOI:** 10.1155/2017/6182492

**Published:** 2017-08-01

**Authors:** Bello Gimba, Saminu Iliyasu Bala

**Affiliations:** ^1^Department of General Studies, School of Health Technology, Jahun, Nigeria; ^2^Department of Mathematical Sciences, Bayero University Kano, Kano, Nigeria

## Abstract

We modeled the impact of bed-net use and insecticide treated nets (ITNs), temperature, and treatment on malaria transmission dynamics using ordinary differential equations. To achieve this we formulated a simple model of mosquito biting rate that depends on temperature and usage of insecticides treated bed nets. We conducted global uncertainty and sensitivity analysis using Latin Hypercube Sampling (LHC) and Partial Rank Correlation Coefficient (PRCC) in order to find the most effective parameters that affect malaria transmission dynamics. We established the existence of the region where the model is epidemiologically feasible. We conducted the stability analysis of the disease-free equilibrium by the threshold parameter. We found the condition for the existence of the endemic equilibrium and provided necessary condition for its stability. Our results show that the peak of mosquitoes biting rate occurs at a range of temperature values not on a single value as previously reported in literature. The results also show that the combination of treatment and ITNs usage is the most effective intervention strategy towards control and eradication of malaria transmissions. Sensitivity analysis results indicate that the biting rate and the mosquitoes death rates are the most important parameters in the dynamics of malaria transmission.

## 1. Introduction

Malaria is one of the most devastating infectious diseases in the world and is caused by* Plasmodium* parasite, which is transmitted via the bites of infected mosquitoes. Among the high risk groups are pregnant women, nonimmune travelers, and children [1]. In pregnant women, malaria has adverse effect on birth outcome which includes low birth weight, abortion, and still born [1]. Apart from health related problems, it also imposes huge socioeconomic burden in malaria-endemic nations. As discussed in Forouzannia and Gumel [2] the annual economic burden of malaria in Africa alone was estimated to be around US $8 billion. These necessitated the formation of several intervention strategies in many countries to mitigate the impact of malaria disease. This includes the use of insecticide treated bed nets (ITNs), intermittent preventive treatment (IPT) especially, for pregnant women during antenatal period, reducing mosquitoes population through the destruction of breeding sites or killing of the larva stage at breading sites that cannot be destroyed [3–5]. Other interventions strategies are the use of indoor residual spraying (IRS) in killing infected mosquitoes resting indoors after blood meal and the use of sterile insect technique [6]. Despite broad efforts for eradication, malaria remains a significant problem resulting in the death of millions of people [2, 7–9]. Most malaria cases and deaths occur in sub-Saharan Africa with Nigeria and Democratic Republic of Congo accounting for about 40% of malaria mortality worldwide [9]. There are a number of characteristics of malaria disease that complicates control efforts. Typical among them is clinical immunity, which is a situation where protection against the clinical symptoms of the disease is developed despite the presence of the parasites [7, 10, 11]. Others include seasonality [12, 13] and treatment failure that might occur due to wrong dosage of medication; see [2] and the references therein. In the context of malaria transmission, seasonality encapsulates complex phenomenon whose definition varies in many studies. Temperature variations have been reported by many to play significant role in the dynamics of malaria transmissions. For example, the report of Roll Back Malaria 2015 indicates that a rise in temperature by 2-3°C will increase the number of people at climatic risk of malaria by 3–5%. Furthermore, the abundance of mosquitoes and the transmission risk have been reported to be influenced by temperature [13–16]. At high temperature, studies have indicated that people are unlikely to use ITNs much [17].

Mathematical models of malaria transmission have been developed by several researchers to gain insight into the dynamics of the disease transmission so as to contribute towards its eradication. Some of these models can be found in [18–23]. These models are in varying degree of complexity. For example, the model of [2] is an age-structured model with several compartments. The model of [7] is made up of four compartments comprising of only the human population. In the model of [5, 17] the authors introduced explicit equation for the proportion of ITNs use as a function of mosquito biting rates.

Some of the problems that complicate malaria control include (1) the presence of individuals who are clinically immune to the disease but can transmit it through bite from susceptible mosquitoes and (2) hot weather which can lead to reduction in the use of ITN. For these reasons, it is important to study the qualitative impact of treatment, immunity, and seasonality on the dynamics of malaria transmission. In this work, we present a vector-host model of malaria transmission dynamics of immune and nonimmune human populations that accounts for the impact of ITN usage and seasonality on the disease. We propose a model of mosquito biting rate as a nonlinear function of temperature and ITN usage to mimic seasonality. This will help in devising optimal intervention strategies that will offer more realistic predictions to control malaria spread. To the best of our knowledge, this is the first vector-host mathematical model for malaria transmission, which explores the impact of daily temperature variations and ITNs usage on control of malaria transmissions. The current study extends the work of [7] by designing a vector-host model for malaria transmission dynamics. The study also extends the work of [17] by modeling the mosquito biting rate as a function of temperature to mimic seasonality. The paper is organized as follows. We formulated the model in Section 2 and analyzed it qualitatively in Section 3, in Section 4 we conducted global uncertainty and sensitivity analysis, Section 5 is the discussion part, and in Section 6 we present our conclusions.

## 2. Model Formulation

In this section, we modify an existing mathematical model for malaria transmission dynamics developed by [7]. The process of the modification is presented below. Following [7] we defined naive individuals as those who have never been infected with malaria, or those who have been infected but have not developed clinical immunity, or those who have lost all immunity. Similarly, clinically immune individuals are those with immunity to clinical symptoms. The total human population denoted by *N*_*h*_ is divided into mutually exclusive subpopulations of susceptible naive *S*_*n*_, susceptible clinically immune *S*_*c*_, infected naive *I*_*n*_, and infected clinically immune *I*_*c*_, so that *N*_*h*_ = *S*_*n*_ + *S*_*c*_ + *I*_*n*_ + *I*_*c*_.

The total mosquitoes population denoted by *N*_*m*_ is divided into compartments of susceptible and infected mosquitoes, so that *N*_*m*_ = *S*_*v*_ + *I*_*v*_.

All recruitment is assumed to be into the susceptible naive human population generated via birth and/or immigration at a rate *λ*_*h*_*N*_*h*_. The population of naive susceptible individuals (*S*_*n*_) is increased by naive infected individuals that recovered without immunity at a rate *γ*_*NN*_, treated naive individuals that recovered without immunity at a rate *ϵ*_*nn*_, and clinically immune individuals that lost immunity at a rate *α*_*c*_. The population of naive susceptible individuals (*S*_*n*_) is decreased by natural death rate *ϕ*_*h*_ = (*μ*_*h*2_*N*_*h*_ + *μ*_*h*_) and force of infection (Λ_*h*_), following effective contacts with infected mosquito. Here *μ*_*h*2_ and *μ*_*h*_ represent the density dependent and density independent part of human death rate and emigration, respectively. We model the force of infection from mosquitoes to human as Λ_*h*_ = *a*(*β*(*T*))*bI*_*v*_/*N*_*h*_.

Here *b* is the probability of infection of susceptible human per bite by an infected mosquito and *a* ≡ *a*(*β*(*T*)) is the biting rate of mosquitoes on susceptible human; *β* ≡ *β*(*T*) represents the proportion of ITN usage and depends on environmental temperature *T*. See (7) for the functional form of *β*. We assumed that temperature is a parameter that is time independent to make the analysis easier. Thus,

(1) dSndt=λhNh−μh2Nh+μhSn+ϵnn+γNNIn+αcSc−ΛhSn.

The clinically susceptible population is generated by the treated naive individuals that become clinically immune, infected naive individuals that recovered with clinical immunity at a rate *γ*_*NC*_, infected clinically immune individuals that recovered with clinical immunity at a rate *γ*_*C*_, and treated clinically immune infected individuals that recover at a rate *T*_*c*_. It is decreased by susceptible clinically immune individuals that lose immunity at rate *α*_*c*_, natural death at rate *ϕ*_*h*_, and the force of infection that pushed out susceptible clinically immune human into infected clinically immune population as a result of contact with infected mosquitoes at rate Λ_*h*_. We assume that clinically infected individuals recover into the clinically susceptible compartment only. Thus,

(2) dScdt=Tn−ϵnn+γNCIn−ΛhSc+Tc+γCIc−αc+μh2Nh+μhSc.

The population of infected naive humans is generated by the population of the infectious susceptible naive human that become infected. It is decreased by the treated infected naive individuals at a rate *T*_*n*_, infected naive individuals that recover without immunity at a rate *γ*_*NN*_, infected naive individuals that recover with immunity at a rate *γ*_*NC*_, the natural death *ϕ*_*h*_, and disease induced death rate *δ*_*N*_. Thus,

(3) dIndt=ΛhSn−Tn+γNN+γNCIn−μh2Nh+μhIn−δNIn.

The population of infected clinically immune human is generated by the population of susceptible clinically immune humans that become infected. It is decreased by the treated infected clinically immune individuals at a rate *T*_*c*_, population of clinically immune individuals that recover with immunity at a rate *γ*_*C*_, the natural death *ϕ*_*h*_, and disease induced death at a rate *δ*_*C*_. Thus,

(4) $$\begin{array}{c} \frac{dI_{c}}{dt}=\Lambda_{h}S_{c}-\gamma_{C}I_{c}-\left(\;\mu_{h2}N_{h}+\mu_{h}\;\right)I_{c}-T_{c}I_{c}-\delta_{C}I_{c}. \end{array}$$

The population of susceptible mosquitoes is generated by birth at a rate *λ*_*v*_. It is reduced by natural death *ϕ*_*v*_ = (*μ*_*v*2_*N*_*m*_ + *μ*_*v*_), contact with ITNs at the rate *βμ*_*v*3_, and infection when in contact with infected human at rate Λ_*v*_ = (*I*_*c*_*τ*_*c*_ + *τ*_*n*_*I*_*n*_)/*N*_*h*_. The parameters *μ*_*v*_ and *μ*_*v*2_ represent the density independent and density dependent parts of the mosquitoes death rate, respectively. Here *τ*_*n*_ = *ac*_1_, *τ*_*c*_ = *ac*_2_, and *c*_1_ and *c*_2_ are the probabilities that susceptible mosquitoes become infectious after biting an infected naive or clinically immune human, respectively. Thus,

(5) dSvdt=λvNm−Icτc+τnInSvNh−μv2Nm+μv+βμv3Sv.

The population of infected mosquitoes is increased by infected susceptible mosquitoes at rate Λ_*v*_. It is decreased by the natural death *ϕ*_*v*_ and death when they come into contact with ITN *βμ*_*v*3_. Thus,

(6) $$\begin{array}{c} \frac{dI_{v}}{dt}=\frac{\left(I_{c}\tau_{c}+\tau_{n}I_{n}\right)S_{v}}{N_{h}}-\left(\;\mu_{v2}N_{m}+\mu_{v}+\beta \mu_{v3}\;\right)I_{v}. \end{array}$$

**Figure 1:**
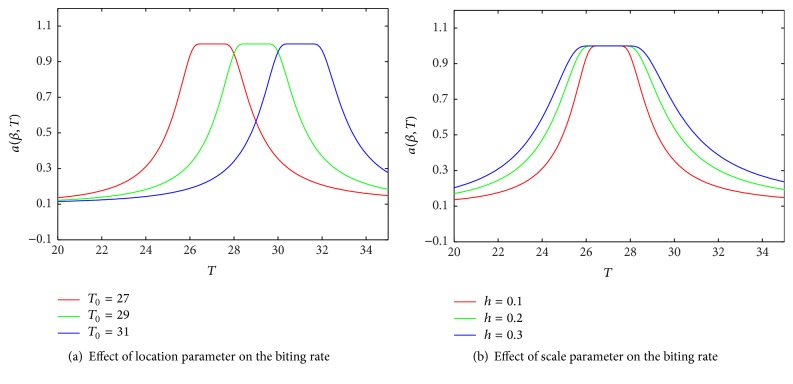
(a) Effects of location parameter on the biting rate for *T*_0_ = 27°C. (b) Effects of scale parameter on the biting rate for *h* = 0.1°C.

In the report of [17], the authors model the mosquitoes biting rate as a linear function of ITN usage while [24] considers a more general form. None of these authors consider the impact of temperature on bed-net use despite its significance. In this work we model the biting rate as

(7) $$\begin{array}{c} a=\beta_{max}-\beta \left(\;\beta_{max}-\beta_{min}\;\right), \end{array}$$

where *β* = *e*^−*h*(*T*/(*T*−*T*_0_))^2^^ represents the proportion of ITN usage and the parameters *T*_0_ and *h* are location and scale parameters measured in °C, respectively. The choice of temperature as a parameter in the biting rate is to mimic seasonality. The justification of this novel approach is due to many reports in literature on the relative importance of temperature in malaria transmission dynamics as outlined in the introduction. In Figure 1, we study impact of shape and scale parameters on the biting rate. From Figure 1, we observe that the optimum temperature for the biting rate is not a single temperature value as discussed in [25] but a range of values. In [15], the authors review some calibrated models of temperature variations in terms of mosquitoes biting rates. One of the findings is that biting rates are optimal at certain temperature values. Typical values reported are 24.4°C, 25.0°C, 26.3°C, and 27.5°C. In this work we are reporting a range of values that encapsulates individual values from several reports. From Figure 1(a), it can be seen that before the maximum biting rate is attained, high values of location parameter will predict relatively lower biting rates. This finding is in contrast to the result of increasing the scale parameter as depicted on Figure 1(b).

**Figure 2:**
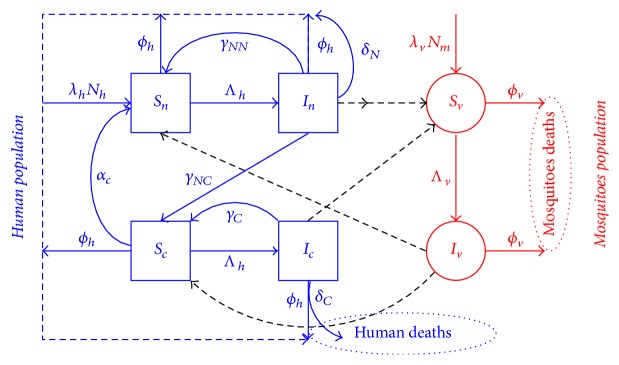
Susceptible humans, *S*_*n*_, *S*_*c*_. can be infected by infectious mosquitoes. They then pass to the respective infectious compartments, *I*_*n*_, *I*_*c*_, before reentering the susceptible classes again or die due to the disease. The susceptible mosquitoes, *S*_*v*_, can become infected when they bite infectious humans. The infected mosquitoes then move to the infectious class *I*_*v*_.

It follows, based on the above derivations and assumptions, that the model for the transmission dynamics of malaria is given by the following deterministic system of nonlinear differential equations. Flow diagram of the model is depicted in Figure 2, and the state variables and parameters of the model are described in Tables 1 and 2, respectively:

(8) dSndt=λhNh−μh2Nh+μhSn+ϵnn+γNNIn+αcSc−abIvSnNh,dIndt=abIvSnNh−Tn+γNN+γNCIn−μh2Nh+μhIn−δNIn,dScdt=Tn−ϵnn+γNCIn−abIvScNh+Tc+γCIc−αc+μh2Nh+μhSc,dIcdt=abIvScNh−γCIc−μh2Nh+μhIc−TcIc−δCIc,dSvdt=λvNm−Icτc+τnInSvNh−μv2Nm+μv+βμv1Sv,dIvdt=Icτc+τnInSvNh−μv2Nm+μv+βμv1Iv,dNhdt=λhNh−μh2Nh+μhNh−δNIn−δCIc,dNmdt=λvNm−μv2Nm+μv+βμv1Nm.

## 3. Model Analysis

### 3.1. Basic Properties of the Model

**Lemma 1 .**

Let *N*_*h*_^*∗*^, *N*_*m*_^*∗*^ be the equilibrium solutions of the total human and mosquito populations, respectively. The closed set *D* = {(*S*_*n*_, *I*_*n*_, *S*_*c*_, *I*_*c*_, *S*_*v*_, *I*_*v*_) ∈ *R*_+_^6^ : *N*_*h*_ ≤ *N*_*h*_^*∗*^, *N*_*m*_ ≤ *N*_*m*_^*∗*^} is positively invariant and attracting.

**Proof**

Adding the first four equations and the last two equations of model (8) we obtained

(9) dNhdt=λhNh−μh2Nh+μhNh−δNIn−δCIc,dNmdt=λvNm−μv2Nm+μv+βμv1Nm.

The mosquito population is modeled by logistic growth with carrying capacity *k*_*v*_ = (*λ*_*v*_ − *μ*_*v*_ − *μ*_*v*1_)/*μ*_*v*2_. It is easy to see that *dN*_*h*_/*dt* ≤ (*λ*_*h*_ − *μ*_*h*_)*N*_*h*_(1 − *N*_*h*_/*k*_*h*_) and *dN*_*m*_/*dt* ≤ (*λ*_*v*_ − *μ*_*v*_)*N*_*m*_(1 − *N*_*m*_/*k*_*v*_). This shows that *dN*_*h*_/*dt* ≤ 0 if 1 ≥ *N*_*h*_/*k*_*h*_ and it approaches *k*_*h*_. Similarly, *dN*_*m*_/*dt* ≤ 0 if 1 ≥ *N*_*m*_/*k*_*v*_ and it approaches *k*_*v*_. Hence, using comparison theory [2]

(10) Nht≤khNh0Nh01−e−λh−μht+khe−λh−μht,Nmt≤kvNm0Nm01−e−λv−μvt+kve−λv−μvt.

If *N*_*h*_(0) ≤ *k*_*h*_, then *N*_*h*_(*t*) ≤ *k*_*h*_ and if *N*_*m*_(0) ≤ *k*_*v*_, then *N*_*m*_(*t*) ≤ *k*_*v*_. Thus, the region D is positively invariant for the model. Moreover, if *N*_*h*_(0) ≥ *k*_*h*_, *N*_*m*_(0) ≥ *k*_*v*_, then either the solution enters the region D in finite time or *N*_*h*_(*t*) → *k*_*h*_, *N*_*m*_(*t*) → *k*_*v*_, as *t* → *∞*. Thus the region attracts all solutions in *R*_+_^6^. Now that we have shown that D is positively invariant, the requirement for existence and uniqueness of solutions holds for the system [2]. The dynamics of the system in the region D will henceforth be investigated.

### 3.2. Scaling

To analyze the malaria model (8), we think it is easier to work with fractional population instead of actual populations by scaling the population of each class by the total species population. We let *S*_*n*_ = *uN*_*h*_, *I*_*n*_ = *vN*_*h*_, *S*_*c*_ = *wN*_*h*_, *I*_*c*_ = *xN*_*h*_, *I*_*v*_ = *zN*_*m*_, *S*_*v*_ = *yN*_*m*_. We arbitrarily scale the time variables by *λ*_*v*_ by introducing *τ* = *tλ*_*v*_ so that

(11) dudτ=1λvNhdSn/dt−SndNh/dtNh2,dvdτ=1λvNhdIn/dt−IndNh/dtNh2

and so on for the rest of the variables. In the absence of the disease, the human population follows logistic growth with carrying capacity *k*_*h*_ = (*λ*_*h*_ − *μ*_*h*_)/*μ*_*h*2_. Following [22] we scaled the human and vector populations in the first 6 equations of model (8) using their respective carrying capacities as *N*_*h*_ = *k*_*h*_*N*_*h*_^*∗*^, *N*_*m*_ = *k*_*v*_*N*_*m*_^*∗*^. So we have a new 6-dimensional system of equations with two additional dimensions for the two total population variables *N*_*h*_ and *N*_*m*_ as

(12) dudτ=−a1bkvNm∗khNh∗z−λu+αw+vγnn+λ,dvdτ=−Tj+γnc1+γnn+λ+δnv+a1bkvNm∗khNh∗uz,dwdτ=−wa1bkvNm∗khNh∗z+α+λ+vTj−ϵn+γnc1+xTi+γc,dxdτ=a1bkvNm∗khNh∗wz−xλ+Ti+γc+δc,dydτ=−vytn−xytc−y+1,dzdτ=vytn+xytc−z,dNhdτ=1−Nh1−ω1Nh−Nhvδn+xδc,dNmdτ=1−Nm1−ω2Nm−Nmω3,

where *λ* = *λ*_*h*_/*λ*_*v*_, *a*_1_ = *a*/*λ*_*v*_, *b*_1_ = *b*/*λ*_*v*_, *α* = *α*_*c*_/*λ*_*v*_, *γ*_*nn*_ = *γ*_*NN*_/*λ*_*v*_, *T*_*i*_ = *T*_*c*_/*λ*_*v*_, *T*_*j*_ = *T*_*n*_/*λ*_*v*_, *ϵ*_*n*_ = *ϵ*_*nn*_/*λ*_*v*_, *δ*_*c*_ = *δ*_*C*_/*λ*_*v*_, *δ*_*n*_ = *δ*_*N*_/*λ*_*v*_, *γ*_*nc*_^1^ = *γ*_*NC*_/*λ*_*v*_, *γ*_*c*_ = *γ*_*C*_/*λ*_*v*_, *t*_*n*_ = *τ*_*n*_/*λ*_*v*_, *t*_*c*_ = *τ*_*c*_/*λ*_*v*_, *ω*_1_ = *μ*_*h*_/*λ*_*v*_, *ω*_2_ = *μ*_*v*_/*λ*_*v*_, and *ω*_3_ = *μ*_*v*3_/*λ*_*v*_.

The analytic solution of model (12) will be more tractable if the number of equations is reduced. To this end, we let *u* = 1 − *v* − *w* − *x*, *y* = 1 − *z*, and by substituting these in (12), two equations are eliminated leaving us with the reduced model:

(13) dvdτ=1−v−w−xξz−Av,dwdτ=−ξz+Fw+mx+vγnc,dxdτ=−Bx+ξwz,dzdτ=−vtn+xtc+1z+vtn+xtc,

where *ξ* = *a*_1_*bk*_*v*_*ρ*/*k*_*h*_, *ρ* = *N*_*m*_^*∗*^/*N*_*h*_^*∗*^, *A* = (*T*_*j*_ + *γ*_*nc*_^1^ + *γ*_*nn*_ + *λ* + *δ*_*n*_), *m* = (*T*_*i*_ + *γ*_*c*_), *B* = (*T*_*i*_ + *γ*_*c*_ + *λ* + *δ*_*c*_), *F* = *α* + *λ*, *γ*_*nc*_ = *T*_*j*_ − *ϵ*_*n*_ + *γ*_*nc*_^1^.

### 3.3. Reproduction Number and Disease-Free Equilibrium

The disease-free equilibrium point (DFE) is the solution of the reduced model (13) when the disease classes are identically zero. By setting the right hand sides of (13) and the disease classes to zero, then solving the resulting equations simultaneously, we obtained the DFE of the reduced model (13) as *E*_dfe_ = (*w*, *v*, *x*, *z*) = (0, 0, 0, 0). The nonnegative matrix for the new infection (*ϝ*) and the matrix for the transition terms (*V*) are given by

(14) ϝ=00ξ000tntc0,V=A000B0001,ϝV−1=00ξ000tnAtcB0.

The spectral radius is

(15) $$\begin{array}{c} \rho \left(\;ϝV^{-1}\;\right)=\sqrt{\frac{\xi t_{n}}{A}}. \end{array}$$

We define the reproduction number as

(16) $$\begin{array}{c} R_{\tau }=\frac{\xi t_{n}}{A}. \end{array}$$

**Lemma 2 .**

The equilibrium point *E*_dfe_ of the reduced model (13) is locally asymptotically stable (LAS) if *R*_*τ*_ < 1 and unstable if *R*_*τ*_ > 1.

**Proof**

The Jacobian matrix evaluated at *E*_dfe_ is

(17) $$\begin{array}{c} J\left(\;E_{dfe}\;\right)=\left[\begin{array}{cccc} -F & \gamma_{NC} & m & 0\\ 0 & -A & 0 & \xi \\ 0 & 0 & -B & 0\\ 0 & t_{n} & t_{c} & -1 \end{array}\right]. \end{array}$$

The characteristic equation is

(18) $$\begin{array}{c} \left(\;F+\eta \;\right)\left(\;B+\eta \;\right)\left(\;\eta \left(\;A+1\;\right)+\eta^{2}-t_{n}\xi +A\;\right)=0. \end{array}$$

The eigenvalues are

(19) η1=−F,η2=−B,η3=−A+1−A+12−4A1−Rτ2,η4=−A+1+A+12−4A1−Rτ2.

Clearly, *η*_*i*_, *i* = 1,2, 3, are negatives and that *η*_4_ is negative provided *R*_*τ*_ < 1.

### 3.4. Endemic Equilibrium Point and Backward Bifurcation

Further analysis of the model is done by finding the endemic equilibrium solutions *x*^*∗*^, *v*^*∗*^, *w*^*∗*^, *z*^*∗*^ of the reduced model (13). First we state the following.

**Theorem 3 .**

The reduced model has the following:

1. A single endemic equilibrium solution if *R*_*τ*_ ≥ 1, or *R*_1_ = 1, and *P*_1_*R*_*τ*_ > *P*_3_.
2. Two endemic equilibrium solutions if *P*_1_*R*_*τ*_ > *P*_3_ and *R*_1_ = 4*P*_2_*P*_4_(1 − *R*_*τ*_)/(*P*_1_*R*_*τ*_ − *P*_3_)^2^ < 1.
3. No endemic equilibrium solution otherwise.

Here *P*_1_ = *A*(*λt*_*n*_ + *γ*_*NC*_*t*_*c*_), *P*_2_ = *A*(*λt*_*n*_ + *γ*_*NC*_*t*_*c*_ + *λ* + *γ*_*NC*_), *P*_4_ = *BFt*_*n*_^2^, and *P*_3_ = *t*_*n*_(*BFt*_*n*_ + *λA* + *BF* + *Bγ*_*NC*_).

**Proof**

To prove this, we first expressed the right hand side of the reduced model (13) in terms of the equilibrium solutions *x*^*∗*^, *v*^*∗*^, *w*^*∗*^, *z*^*∗*^. We then eliminated the rest of the variables leaving only one equation in terms of *z*^*∗*^ as

(20) z∗2ξ2z∗2λtn+γNCtc+λ+γNC+z∗−λtn−γNCtc+BFtn+λA+BF+γNCzi∗−BFtnξ+ABF=0.

This equation can be written in terms of *R*_*τ*_ as

(21) $$\begin{array}{c} P_{2}{R_{\tau }}^{2}{z^{*}}^{2}+\left(\;-P_{1}{R_{\tau }}^{2}+P_{3}R_{\tau }\;\right)z^{*}-P_{4}R_{\tau }+P_{4}=0. \end{array}$$

The nonzero solutions of (21) can be written as

(22) $$\begin{array}{c} z_{i}^{*}=\left(\;\frac{P_{1}R_{\tau }-P_{3}}{2P_{2}R_{\tau }}\;\right)\left(\;1\pm \sqrt{1-\frac{4P_{2}P_{4}\left(1-R_{\tau }\right)}{{\left(P_{1}R_{\tau }-P_{3}\right)}^{2}}}\;\right). \end{array}$$

It can be seen from (22) that, for *R*_*τ*_ > 1, *z*_*i*_^*∗*^ can only have one positive value. We expressed the other variables in terms of *z*_*i*_^*∗*^ as

(23) xi∗=zi∗2ξγNC1−zi∗λξzi∗tn+ξzi∗γNCtc+BFtn,vi∗=λξzi∗+BFzi∗1−zi∗λξzi∗tn+ξzi∗γNCtc+BFtn,wi∗=Bzi∗γNC1−zi∗λξzi∗tn+ξzi∗γNCtc+BFtn.

It is clear from (23) that *x*_*i*_^*∗*^, *v*_*i*_^*∗*^, *w*_*i*_^*∗*^ are positive whenever *z*_*i*_^*∗*^ is. Hence, the endemic equilibrium point exists when *R*_*τ*_ > 1 and is uniquely given by *E*_*i*_ = (*x*_*i*_^*∗*^, *v*_*i*_^*∗*^, *w*_*i*_^*∗*^, *z*_*i*_^*∗*^), *i* = 1  or  2.

Now suppose *P*_1_*R*_*τ*_ − *P*_3_ > 0 and *R*_1_ < 1. It is clear from (22) that both values of *z*_*i*_^*∗*^ are positive. In this case, the equilibria can be written as *E*_*e*_ = (*x*_*i*_^*∗*^, *v*_*i*_^*∗*^, *w*_*i*_^*∗*^, *z*_*i*_^*∗*^), *i* = 1,2.

This establishes the second part of Theorem 3. It can easily be seen from (22) that the other possibilities *R*_1_ > 1, or *R*_1_ = 1, *P*_1_*R*_*τ*_ < *P*_3_, or *R*_1_ < 1, *P*_1_*R*_*τ*_ < *P*_3_ will not result in any real positive value of *z*_*i*_^*∗*^.

Backward bifurcation, which is a situation where the locally asymptotically stable DFE coexists with a locally asymptotically stable endemic equilibrium point, has been observed in many epidemic models. See, for instance, [2, 7, 28, 29]. In this scenario, the requirement for the basic reproduction number to be less than one for the disease to be eradicated no longer holds. Condition (2) of Theorem 3 provides possibility of backward bifurcation in our model. Here we provide parameter range within which backward bifurcation is likely to happen. To do this, it is enough to show that there is a positive endemic equilibrium when the basic reproduction number *R*_*τ*_ < 1. We state the result in the following lemma.

**Lemma 4 .**

The reduced model (13) undergoes backward bifurcation when *P*_3_/*P*_1_ < *R*_*τ*_^*b*^ < *R*_*τ*_ ≤ 1, where

(24) Rτb=P1P3−2P2P4+2P12P2P41−P3/P1+P22P42P12.

To prove this, we first solve for the value of *R*_*τ*_ such that (*P*_1_*R*_*τ*_ − *P*_3_)^2^ + 4*P*_2_*P*_4_(*R*_*τ*_ − 1) = 0, where we obtained two possibilities as

(25) Rτb=P1P3−2P2P4+2P12P2P41−P3/P1+P22P42P12,Rτ1=P1P3−2P2P4−2P12P2P41−P3/P1+P22P42P12.

Using condition (2) of Theorem 3 and (25), backward bifurcation will occur if *R*_*τ*_ < 1 and *R*_*τ*_ > max{*P*_3_/*P*_1_, *R*_*τ*_^*b*^, *R*_*τ*_^1^}. Now it is clear that *R*_*τ*_^*b*^ > *R*_*τ*_^1^. Now consider the difference

(26) Rτb−P3P1=2−P2P4+P12P2P41−P3/P1+P22P42P12>0.

In analogous fashion we show that *P*_3_/*P*_1_ > *R*_*τ*_^1^. Hence we get the result.

### 3.5. Stability of the Unique Endemic Equilibrium

Under the hypothesis of condition (1) of the theorem, the endemic equilibrium point of the reduced model (13) exists and is unique. Some of its local stability properties can now be investigated. Without loss of generality, we assume that the endemic equilibrium point is *E*_1_ = (*x*_1_^*∗*^, *v*_1_^*∗*^, *w*_1_^*∗*^, *z*_1_^*∗*^) and we dropped the asterisks. The local stability of *E*_1_ is now investigated by linearizing the right hand side of model (13) about the equilibrium solution. This gives the Jacobian matrix

(27) JE1=−ξz1−FγNCm−ξw1−ξz1−ξz1−A−ξz1−ξv1−ξw1−ξx1+ξξz10−Bξw10−tnz1+tn−tcz1+tc−tcx1−tnv1−1.

The characteristic polynomial is

(28) $$\begin{array}{c} \eta^{4}+A_{3}\eta^{3}+A_{2}\eta^{2}+A_{1}\eta +A_{0}=0, \end{array}$$

where

(29) A3=v1tn+x1tc+2ξz1+A+B+F+1,A2=z1−11−v1−x1−w1tn+z1+2v1z1tnξ+A+B+Fv1tn+2x1ξz1+A+B+Fx1tc+z1−1w1tc+z1ξ+ξ2z12+A+B+F+λ+γNCξz1+AB+AF+BF+A+B+F,A1=−z1+1ξ2z1+ξK1w1+x1tn+Fξz1−1w1tc+tn+z1+ξBz1+z1−1tn+z1ξ2z1−1w1tc+tn+z1+Aξz1−1w1tc+z1+ξ2z12+B+K2+λ+γNCξz1+K1A+BF·x1tc+ξ2z1+A+λ+γNCz1+K1ξ+K1A+BF·v1tn+λ+γNCξ2z12+Aλ+BF+BγNC+λ+γNCξz1+BF+K1A+BF,A0=Rτz1P1Rτ+P4ART−11−z1tn2,K1=B+F,K2=A+F,RT=z1RτQ1+P42Rτz1P1Rτ+P42,

*Q*_1_ = *z*_1_(*P*_3_*R*_*τ*_ + *P*_4_)*P*_1_ + *z*_1_(−2*z*_1_ + 3)*P*_2_*P*_4_*R*_*τ*_ + (−*z*_1_ + 2)(*z*_1_^2^*P*_1_*P*_2_*R*_*τ*_^2^ + *P*_3_*P*_4_). Notice that *A*_3_, *A*_2_, *A*_1_ are all positives and *A*_0_ can be positive or negative depending on the sign of (*R*_*T*_ − 1). Hence, by Hurwitz criterion, for *E*_1_ to be locally stable, it is necessary that *R*_*T*_ > 1.

### 3.6. Effects of ITNs Use and Temperature on the Basic Reproduction Number

We evaluate the effects of ITNs use and temperature on the dynamics of malaria on the reduced model (13) through their elasticity indices *R*_*τ*_^*β*^ = (*β*/*R*_*τ*_)(∂*R*_*τ*_/∂*T*) and *R*_*τ*_^*T*^ = (*T*/*R*_*τ*_)(∂*R*_*τ*_/∂*β*), respectively; see [24, 26] for more explanation on this procedure. The analytical formulations of the elasticity indices of the proportion of ITNs use and temperature with respect to *R*_*τ*_ are given, respectively, by (30) and (31).

(30) Rτβ=2βαβmax−βminαβmax−βmin−βmax<0,

(31) RτT=2ThT+T0e−ζβmax−βminT−T03e−ζβmax−βmin−βmax,T≠T0,

where *ζ* = *hT*/(*T* − *T*_0_)^2^. The implication of (30) is that an increase in the number of ITNs used will bring about decrease in the reproduction number and vice versa. This finding corroborates the report of [24]. From (31), the reproduction number will increase with temperature whenever *T* < *T*_0_ and will decrease with temperature whenever *T* > *T*_0_. In other words, the reproduction number is not a monotonic function of temperature. It should be understood that this type of sensitivity analysis only focuses on specific parameters. It does not indicate the effect of concurrent, large perturbations in all model parameters which is almost always the case in epidemiology. In order to gain more insight into the sensitivities of the conglomerations of the parameters, we carry out more investigations in the next section.

## 4. Global Uncertainty and Sensitivity Analysis

**Figure 3:**
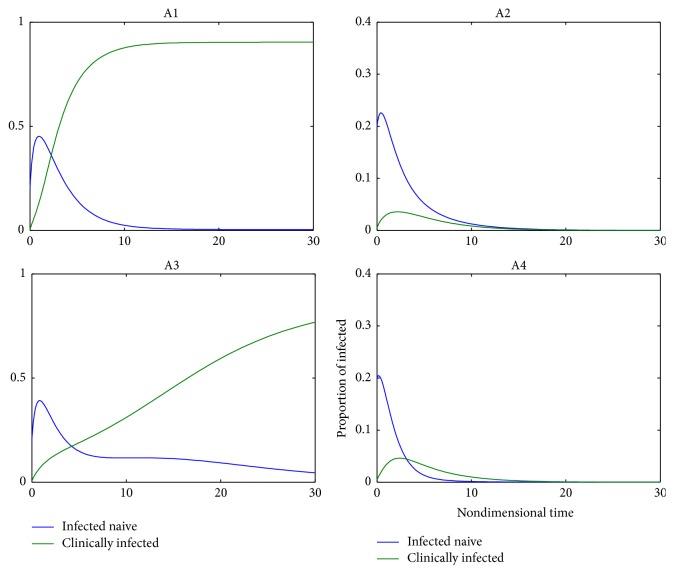
Time course of the reduced model with initial condition (*w*, *v*, *x*, *z*) = (0.1,0.2,0.005,0.3). Panel A1 corresponds to case *R*_*τ*_ = 4.0573, *R*^*b*^*τ* = 0.3920. Panel A2 corresponds to case *R*_*τ*_ = 0.5341, *R*^*b*^*τ* = 0.9952. Panel A3 corresponds to case *R*_*τ*_ = 0.3397, *R*^*b*^*τ* = 0.0901. Panel A4 corresponds to case *R*_*τ*_ = 0.092, *R*^*b*^*τ* = 0.4143. The legend and labels on A4 are the same for all the subfigures.

**Figure 4:**
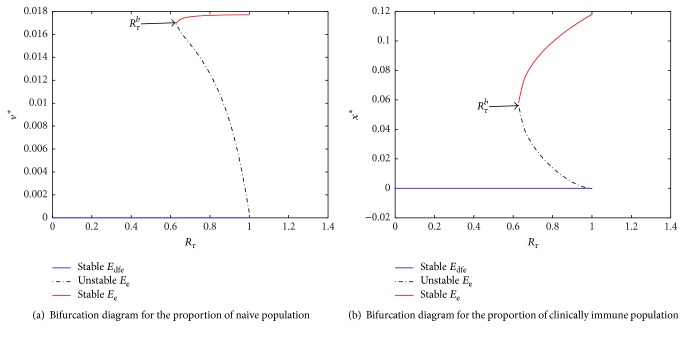
(a) Proportion of infected naive population. (b) Proportion of clinically infected population.

**Figure 5:**
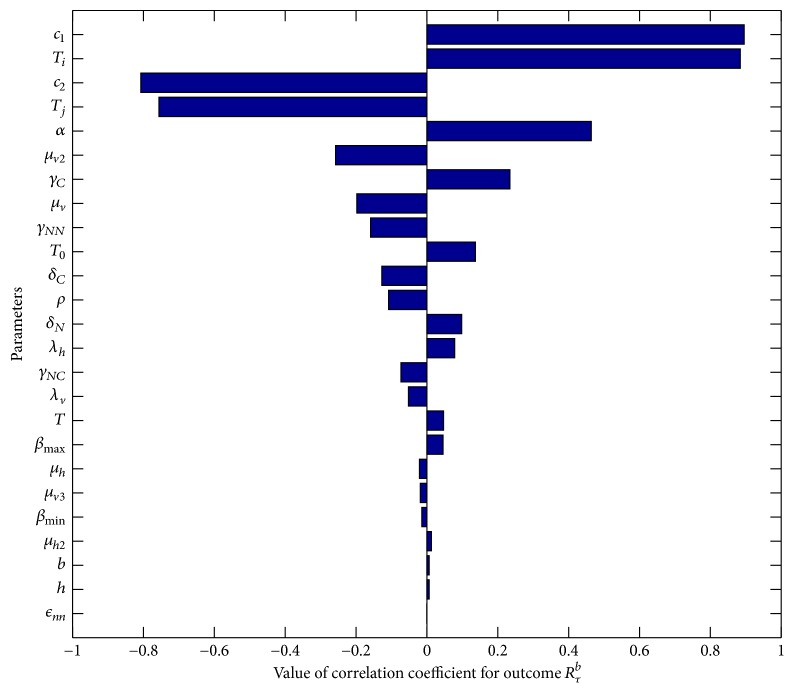
Tornado plot of sensitivity analysis of the bifurcation parameter *R*_*τ*_^*b*^.

**Figure 6:**
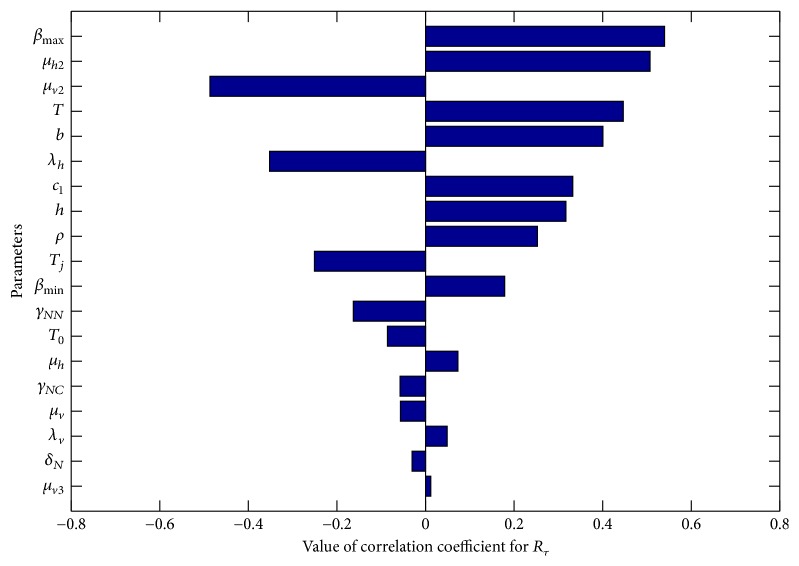
PRCC results for all the parameters affecting the threshold parameter *R*_*τ*_.

One of the important components of epidemic modeling is parameter estimation. This is because many factors combine to form variability in inputs into the model output. These factors may include erroneous parameter estimation and uncertainty in the exact parameter values. For this reason, it is important to determine parameters that have substantial influence on the results. This can be achieved through sensitivity and uncertainty measurement that can be done more easily due to rapid growth in computing technology. For example, Sampling and Sensitivity Analysis Tools (SaSAT) is software developed for sensitivity analysis [30]. In our model, the proportion of infectious human population, *v*, *x*, are regulated by various malaria-related epidemiological parameters that are shrouded with uncertainty. Following [30], we use the Latin Hypercube Sampling (LHS) and Partial Rank Correlation Coefficient (PRCC) techniques to perform a global uncertainty and sensitivity analysis of the reduced model (13). We sample all the twenty-five parameters of the model and then carry out simulations to measure their statistical influence on the proportion of infectious humans *v*, *x*. Using published results in literature, we assigned upper and lower bounds for each parameter as shown on Table 3. As in [24, 30], we assumed that each parameter follows a uniform distribution and then draw 1000 samples from the distribution. This gives a 1000 × 25 matrix whose rows consist of unique collections of parameters. For each row of the matrix, the reduced model (13) is integrated using MATLAB ode45 and we record the proportion of infected humans at each time step. Typical results of such calculations are depicted on Figure 3. The legend and the axes labels on A4 are the same for all the subfigures. From Panel A1 of the figure *R*_*τ*_ > 1 and there is no bifurcation. This implies that the disease will invade and will persist. In this case, it is possible to eradicate the disease by using any intervention that will decrease the reproduction number to below unity. Panels A2 and A4 depict the scenario *R*_*τ*_ < 1 and there is no bifurcation. This means that the disease will not persist and will not invade. In Panel A3 we have typical scenario where the endemic equilibrium point and the DFE coexist. In this case, the disease will persist but will not invade. The results suggest that it is the presence of the immune individuals that makes the disease persist. The strategy that will be adopted here will be examined through sensitivity analysis. From the 1000 collections of unique parameters, 469 satisfy the condition for backward bifurcation. We selected one of these collections as our baseline values as shown on Table 3 except for *T*_*i*_. We conducted sensitivity analysis on the bifurcation parameter using PRCC with the baseline values. To achieve this, we set *T*_*i*_ = 0.5532 and allow *c*_1_ to vary in the range [0.2299,0.35]. The results are depicted on Figure 4. The existence of backward bifurcation implies that the classical requirement for *R*_*τ*_ < 1 for an epidemic to be eradicated no longer holds. The region to the left of the bifurcation point represents the area where malaria will not invade and will not persist (comfort zone), whereas the region enclosed by the solid red and the dash-dotted curves and *R*_*τ*_ = 1 represent the area where malaria will persist but will not invade. If malaria is to be eradicated, the comfort zone should be maximized subject to *R*_*τ*_ ≤ 1 or we must have *R*_*τ*_ < *R*_*τ*_^*b*^. The natural question is what are those parameters that are the most important to the bifurcation parameter? To investigate this, we conducted sensitivity analysis on the bifurcation parameter using the 469 samples mentioned earlier. The results of this analysis are depicted on Figure 5. From the figure, the parameters *c*_1_, *T*_*i*_,  *c*_2_, *T*_*j*_ are the most sensitive to the bifurcation parameter in that order, while in decreasing order of importance the following have the least significance in terms of bifurcation; *β*_min_, *μ*_*h*2_, *b*, *h*, and *ϵ*_*nn*_. Increasing *c*_1_ and *T*_*i*_ will bring about increase in the bifurcation parameter. The public health implication of this result is that increase in treatment of the clinically immune individuals will increase the comfort zone. However, parameter sensitivities on bifurcation parameter should not be taken in isolation. Thus, we draw new 1000 samples for all the 19 parameters affecting *R*_*τ*_ and using these samples, we carry out sensitivity analysis on *R*_*τ*_ using LHS and PRCC. The results of these analyses are depicted on Figure 6. We list the following parameters in order of their importance on *Rτ*, *β*_max_, *μ*_*h*2_, *μ*_2_, and *T* from Figure 6, while the following have least importance and are listed in decreasing order of importance: *μ*_*v*_, *λ*_*v*_, *δ*_*N*_, and *μ*_*v*3_.

### 4.1. Intervention Strategies

**Figure 7:**
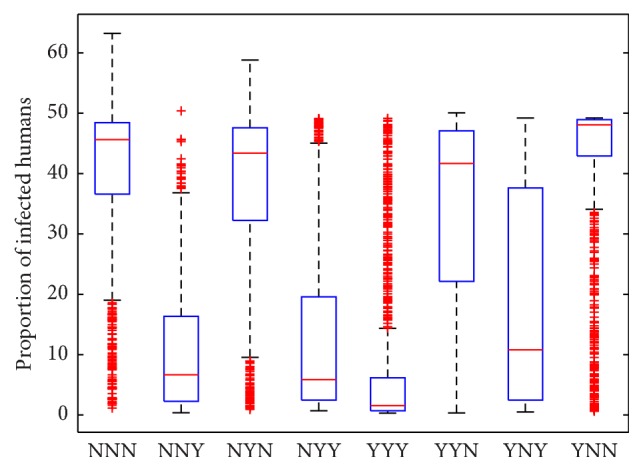
Box plots showing the sum of proportion of infected human populations subjected to different intervention strategies.

**Figure 8:**
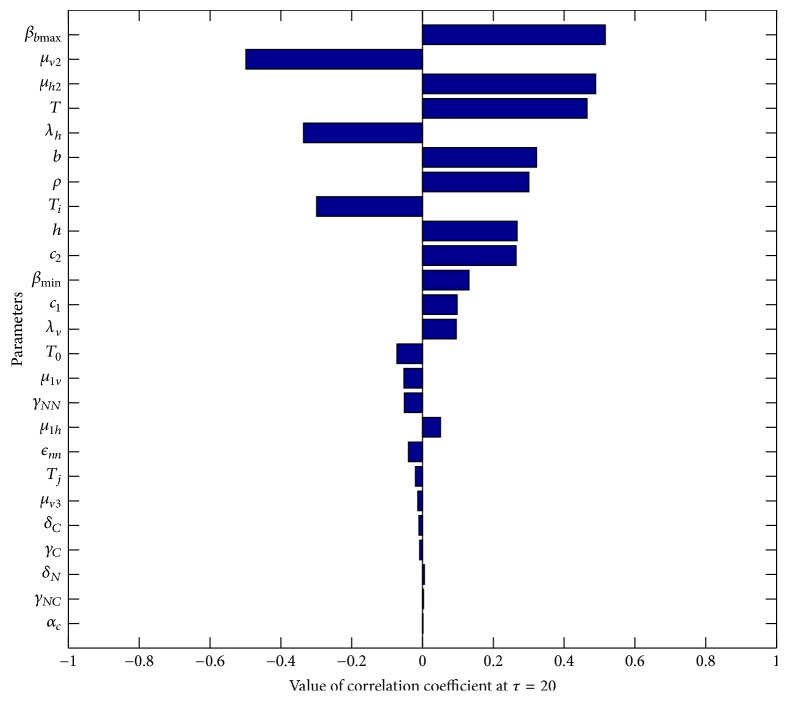
PRCC results for all the parameters of the reduced model, simulated at time *τ* = 20.

**Figure 9:**
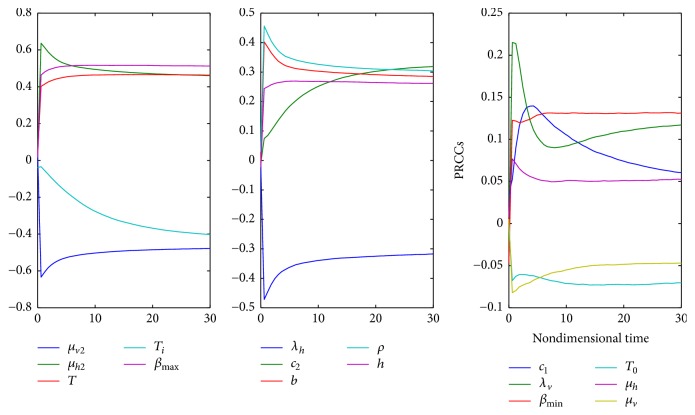
Time dependent PRCC results for 16 parameters of the model. We can infer from the figure that the maximum biting rate and the mosquitoes death rate have the most significant impact in the long run.

**Figure 10:**
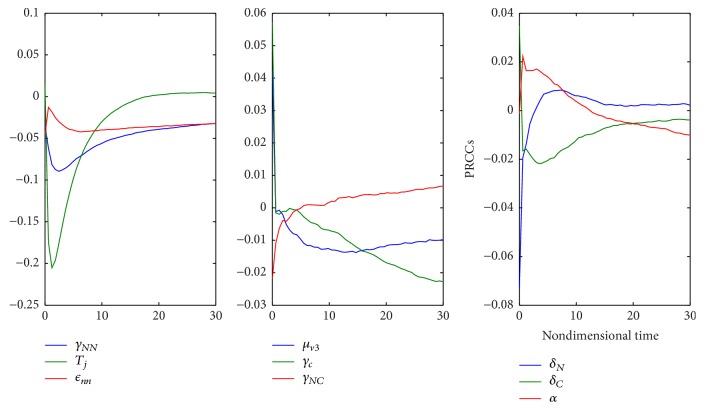
Time dependent PRCC results for 9 parameters of the model. We can infer from the figure that treatment rate for naive infected individuals can have both positive and negative values of PRCCs.

In our model, there are many biological parameters that influence disease dynamics. Also, there are intervention parameters that are crucial for eliminating an epidemic. These are *β*, *T*_*i*_, *T*_*j*_. We consider a range of different intervention strategies by taking 1000 samples using LHS for 19 parameters of the model. The intervention parameters were not sampled but are given specific values as shown on Table 4. Similarly, *T*, *T*_0_, and *h* were not sampled because *β* is constant for a given run of simulation. We used MATLAB ode45 suite to integrate the reduced system (13) using the initial condition: (*w*, *v*, *x*, *z*) = (0.1,0.2,0.005,0.3). For each run of simulation, we recorded the sum of proportions of infected human over the entire time steps. The results of these intervention strategies are presented in the form: NNN ≡ *T*_*j*_ = 0, *T*_*i*_ = 0, *β* = 0; NNY ≡ *T*_*j*_ = 0, *T*_*i*_ = 0, *β* = 0.9; NYN ≡ *T*_*j*_ = 0, *T*_*i*_ = 0.2, *β* = 0, and so forth. The first means no treatment of both the naive and clinically infected populations and no ITN coverage. The second means coverage, no treatment of both the naive and clinically infected populations with 90% ITN coverage. Altogether there are 8 different interventions which correspond to 8000 simulations. The box plot of the results is depicted on Figure 7. The strategy YYY appears to be the best. It skews to the right and is characterized by low median and small interquartile range and the presence of large upper outliers. The strategies NNY and NYY are the better options after YYY. These further demonstrate the effectiveness of ITNs usage and that any intervention with *∗∗*N (any intervention that does not constitute ITNs coverage) is relatively ineffective. The public health interpretation of this result is that reducing contacts between humans and mosquitoes is important in controlling the size of the infectious human population. We conducted further sensitivity analysis by integrating the time dependent dynamics of (13) using 1000 parameter sets used in plotting Figure 3 and the same initial conditions. To do that, the nondimensional time [0,30] was divided into 50 times steps and we recorded the number of infected humans at each time step. The results of these analyses are shown on Figure 8 for the nondimensional time *τ* = 20. The PRCC for *γ*_*NN*_ is the largest and positive, which indicates that rate of recovery without immunity can increase the number of infected humans. The PRCC for *δ*_*C*_ is the second largest in magnitude and has negative sign. This indicates that death rate of clinically immune individuals will decrease the number of infectious humans. The parameter *β*_min_ has the least importance. The PRCCs results depicted on Figure 8 correspond to a single time step; however, the dynamics of the PRCCs is likely to vary over time. Hence, to fully characterize how sensitive the infectious human population is to the parameters of the reduced system (13), we look into the evolution of the PRCCs over time and the results are depicted on Figures 9 and 10 for 0 ≤ *τ* ≤ 30. We grouped the parameters in the figures for the purpose of clarity of presentation only. The results on these figures indicate that, initially, *μ*_*h*2_ and *μ*_*v*2_ are the most significant parameters. While increase in *μ*_*v*2_ will result in decreasing the number of infected humans, increase in *μ*_*h*2_ will lead to increase in the number of infected human. See panel 1 on Figure 9. The maximum biting rate *β*_max_ and the density dependent part of mosquitoes death rate appear to be the most important parameters in the long run. The public health implication of this is that increased ITNs coverage and death rate of mosquitoes can lead to the control of malaria transmission.

## 5. Discussion

Mosquitoes are very efficient vectors of human diseases and are responsible for transmitting some of the many devastating diseases today. For many of these diseases, climate change is key in determining the ability of a mosquito to transmit the disease through the population effectively. One of such diseases is malaria, which is widely considered as the most devastating and the most prevalent human vector borne disease, with one-half of the world population living in areas where there is risk of infection [14]. Two of the factors that complicate malaria control are as follows.

1. Seasonal changes: This has strong influence on malaria transmission which makes it difficult to predict future malaria intensity accurately. There is no single definition of seasonality in relation to malaria in the literature. Malaria metrics such as the mosquito biting rates have been investigated in many studies in relation to temperature; see for instance [15]. Numerous values of mosquitoes biting rates have been reported in literature. In many studies, the biting rate has constant values and these values are mostly different. Given the importance of biting rate as a driving force for malaria transmission, there is the need to conduct further studies on this so as to discern the factors that bring about changes in the biting rate. Typical of these factors is seasonality. This is because transmission rate of malaria has been reported to peak in certain period of the year. It is quite reasonable to model the biting rates as a function of temperature in order to mimic seasonality.
2. Clinical immunity: Another factor that complicates malaria control is that individuals living in regions where malaria is endemic can develop immunity to malaria which enables them to remain asymptomatic while still carrying the parasites [7, 31]. The development of acquired clinical immunity by individuals will result in such individuals not seeking treatment for a long time. Thus, they will harbor the disease and can transmit it when bitten by mosquitoes.

From Panels A1 and A3 of Figure 3, the infectivity of the clinically infected individuals does not wane with time. This might be partly attributed to the reason outlined in bullet (2) above. In view of this finding, one may pause the question; how do we control malaria without treatment? We believe that treatment is inevitable once malaria cases are established. In this case one can consider a number of other intervention strategies that can be combined together with treatment towards control and eradication of malaria transmission. Modeling the dynamics of malaria by separating human population into immune and nonimmune classes, incorporating the temperature and ITNs use as part of mosquitoes biting rates and sensitivity analysis is the main focus of this paper.

Our simple model of temperature dependent biting rate generalizes calibrated model results reviewed by [15]. The local sensitivity analysis indices results are in line with literature reports. For instance, it is common knowledge that the use of fan in our houses could reduce contact between human and mosquitoes and this can lead to reduction in transmission of malaria. The local sensitivity analysis of the reproduction number in relation to temperature results also supports this observation and other similar findings in many reports. See [15] and the references therein.

To eradicate malaria in bistable regions, we need to make *R*_*τ*_^*b*^ > *R*_*τ*_. This can be achieved by considering strategies that will decrease *R*_*τ*_ or increase *R*_*τ*_^*b*^ sufficiently. The sensitivity analysis results reveal that we need to embark on combination of strategies that will decrease the possibilities of immune individuals becoming infective and treating them in order to effectively reduce *R*_*τ*_. This is quite difficult because clinically immune population are not likely to seek treatment for a long time. Moreover, failing to detect the parasite in them may not necessarily mean the absence of the disease. In view of these difficulties, the best thing to do is to embark on maximum ITNs usage. Is there any way in which the rate at which immune individuals lose their clinical immunity can be accelerated? if so, then it will help towards increasing *R*_*τ*_^*b*^ and hence malaria eradication in a bistable region. Other metrics that will assist significantly in this direction are the increase in mosquitoes death rates and temperature. In some African countries such as Nigeria where malaria is endemic and electricity supply is erratic, improved supply of electricity will enable people to use fans and air conditioning system to provide relatively colder environment in their houses. It will also help to provide enabling environment for effective usage of ITNs and also reduces human-mosquitoes contact.

In general, the sensitivities of the model parameters are time dependent. In other words, the most sensitive parameter of the model at the onset of the epidemic may not necessarily maintain the same status at later times. Of particular interest is the fact that the maximum biting and death rates of mosquitoes are the most important parameters in the long run. The public health implication of this is that combined effort should be put in place in reducing human-mosquitoes contact and reduction of mosquito population by any means possible.

## 6. Conclusion

In this paper, we formulated a deterministic model of malaria transmission by dividing the human population broadly according to whether an individual is immune to clinical symptoms of malaria or not. We proposed a simple model with mosquitoes biting rate that is temperature dependent so as to mimic seasonality. We investigated stability conditions for the disease-free equilibrium point and we have found the necessary condition for the unique endemic equilibrium point to be locally stable. We have also shown the range of parameter values in which backward bifurcation can occur. We conducted global sensitivity analysis using Latin Hypercube Sampling and Partial Rank Correlation Coefficient in order to identify the most important parameters that govern the dynamics of malaria transmissions.

We find that the combination of treatment and usage of insecticide treated net is the most effective strategy for malaria control. Provision of relatively colder environment will positively impact malaria control. Mosquitoes biting and death rates are the most important parameters of malaria transmissions. The peak of mosquitoes biting rates occurs not at a single temperature value but as a range of values. Temperature is positively correlated to the reproduction number. The disease-free equilibrium point of the model is locally asymptotically stable when the reproduction number is less than one and unstable when it is greater than one.

## Figures and Tables

**Table 1:** Description of state variable of the model.

Variable	Description
*S*_*n*_	Population of susceptible naive human
*I*_*n*_	Population of infected naive human
*S*_*c*_	Population of susceptible clinically immune human
*I*_*c*_	Population of infected clinically immune human
*N*_*h*_	Total human population
*S*_*v*_	Population of susceptible mosquitoes
*I*_*v*_	Population of infected mosquitoes
*N*_*m*_	Total population of mosquitoes

**Table 2:** Parameters and their descriptions.

Parameters	Description and dimension
*λ*_*h*_	Recruitment rate into human population (humans × day^−1^)
*λ*_*v*_	Recruitment rate into mosquitoes population (mosquitoes × day^−1^)
*T*_*n*_	Treatment rate of infected naive humans (day^−1^)
*T*_*c*_	Treatment rate of clinically infected immune humans (day^−1^)
*c*_1_	Probability of transmission of infection from infected naive human to susceptible mosquito
*c*_2_	Probability of transmission of infection from infected immune human to susceptible mosquito
*ρ*	Number of mosquitoes per human host
*b*	Probability of transmission of infection from an infected mosquito to susceptible human
*T*	Temperature (°C)
*T*_0_	Location parameter (°C)
*β*_max_	Maximum biting rate per mosquito (day^−1^)
*β*_min_	Minimum biting rate per mosquito (day^−1^)
*h*	Scale parameter (°C)
*ϵ*_*nn*_	Rate at which treated naive individuals recovered without immunity (day^−1^)
*γ*_*NN*_	Rate at which naive individuals recovered without immunity (day^−1^)
*γ*_*NC*_	Rate at which naive individuals recovered with clinical immunity (day^−1^)
*α*_*c*_	Rate at which clinically susceptible individuals lose immunity (day^−1^)
*δ*_*N*_	Disease induced death rate for a naive individual (day^−1^)
*δ*_*C*_	Disease induced death rate for a clinically immune individual (day^−1^)
*γ*_*C*_	Recovery rate for clinically immune individuals (day^−1^)
*μ*_*h*2_	Density dependent part of the death and emigration rate for humans (human day^−1^)
*μ*_*h*_	Density independent part of the death rate for humans (human day^−1^)
*μ*_*v*_	Density independent part of the death rate for mosquitoes (mosquitoes day^−1^)
*μ*_*v*2_	Density dependent part of the death rate for mosquitoes (day^−1^)
*μ*_*v*3_	ITN induced death rate for mosquitoes (day^−1^)

**Table 3:** Parameters, their baseline values, and the ranges for sensitivity analysis.

Parameter	Baseline value	Range	Reference
*λ*_*h*_	9.3614×10^−5^	[2.7,14] × 10^−5^	[26]
*λ*_*v*_	0.4478	[0.27,0.7]	[8, 26]
*T*_*n*_	0.1201	[0.03704,0.4]	[21]
*T*_*c*_	0.0476	[0.003704,0.2]	[21]
*c*_1_	0.2299	[0.1,0.7]	[24]
*c*_2_	0.5342	[0.072,0.64]	[24]
*ρ*	7	[2,8]	[27]
*b*	0.8	[0.1,0.8]	[8, 26]
*T*	22	[20,35]	Estimated
*T*_0_	30.6	[27,31]	Estimated
*β*_max_	0.6334	[0.1,1]	[24]
*β*_min_	0.0696	[0,0.1]	[24]
*h*	0.3165	[0.1,0.5]	Estimated
*ϵ*_*nn*_	0.2201	[0.1,0.5]	Estimated
*γ*_*NN*_	0.0805	[0.056,0.2]	[24]
*γ*_*NC*_	0.0106	[0.0014, 0.017]	[7, 24]
*α*_*c*_	0.0022	[55 × 10^(−6)^, 11 × 10^(−3)^]	[7, 24]
*δ*_*N*_	5.7341 × 10^−5^	[0,4.1] × 10^−4^	[26]
*δ*_*C*_	3.2084 × 10^−4^	[0,4.1] × 10^−4^	[26]
*γ*_*C*_	0.0101	[0.0014,0.017]	[7, 24]
*μ*_*h*2_	6.0146 × 10^−7^	[1,100] × 10^−8^	[26]
*μ*_*h*_	1.6728 × 10^−5^	[1,20] × 10^−6^	[26]
*μ*_*v*_	0.0668	[1,100] × 10^−3^	[26]
*μ*_*v*2_	6.8754 × 10^−4^	[1,1000] × 10^−6^	[26]
*μ*_*v*3_	0.0995	[0.0476,0.1]	[24]

**Table 4:** Parameter values used for interventions.

Parameter/symbolic representation	N	Y
*T*_*j*_	0	0.5
*T*_*i*_	0	0.2
*β*	0	0.9
